# Health examination utilization in the visually disabled population in Taiwan: a nationwide population-based study

**DOI:** 10.1186/1472-6963-13-509

**Published:** 2013-12-05

**Authors:** Yueh-Han Hsu, Wen-Chen Tsai, Pei-Tseng Kung

**Affiliations:** 1 Department of Health Services Administration, China Medical University, Taichung 40402, R.O.C. Taiwan; 2 Department of Internal Medicine, Ditmanson Medical Foundation Chia-Yi Christian Hospital, Chia-Yi 60002, R.O.C. Taiwan; 3 Department of Nursing, Min-Hwei Junior College of Health Care Management, Tainan 73658, R.O.C. Taiwan; 4 Department of Healthcare Administration, Asia University, No. 500 Lioufeng Road, Wufeng, Taichung 41354, R.O.C. Taiwan

**Keywords:** Disability, Visual impairment, Health examination utilization, Health disparity

## Abstract

**Background:**

People with visual disabilities have increased health needs but face worse inequity to preventive health examinations. To date, only a few nationwide studies have analyzed the utilization of preventive adult health examinations by the visually disabled population. The aim of this study was to investigate the utilization of health examinations by the visually disabled population, and analyze the factors associated with the utilization.

**Methods:**

Visual disability was certified by ophthalmologists and authenticated by the Ministry of the Interior (MOI), Taiwan. We linked data from three different nationwide datasets (from the MOI, Bureau of Health Promotion, and National Health Research Institutes) between 2006 and 2008 as the data sources. Independent variables included demographic characteristics, income status, health status, and severity of disability; health examination utilization status was the dependent variable. The chi-square test was used to check statistical differences between variables, and a multivariate logistic regression model was used to examine the associated factors with health examination utilization.

**Results:**

In total, 47,812 visually disabled subjects aged 40 years and over were included in this study, only 16.6% of whom received a health examination. Lower utilization was more likely in male subjects, in those aged 65 years and above, insured dependents and those with a top-ranked premium-based salary, catastrophic illness/injury, chronic diseases of the genitourinary system, and severe or very severe disabilities.

**Conclusion:**

The overall health examination utilization in the visually disabled population was very low. Lower utilization occurred mainly in males, the elderly, and those with severe disabilities.

## Background

People with disabilities have distinct healthcare needs, and they tend to experience chronic health problems earlier than the general population [1]. The prevalence of chronic disease is two to three times higher in people with disabilities [2], and the risk of co-morbidities such as cardiovascular disease and stroke is also increased [1,3]. However, previous research has demonstrated that the health service needs of people with disabilities are not currently being met [4-6]. The visually disabled population, as with individuals with other disabilities, have less access to appropriate healthcare services [1,7] and are less likely to receive screening examinations [8]. They face enormous barriers in accessing proper preventive healthcare, including informational barriers, lack of services, lack of transport, inadequate resources or financial considerations, lack of social awareness, and lack of education and training of healthcare providers [9-11]. In terms of equity, we hypothesized that the visually disabled are being doubly marginalized.

Visual impairment is one of the major causes of disability in the United States and in Taiwan [12,13]. It has been estimated that the prevalence of visual disabilities will increase markedly during the next several decades, with an estimated 70% increase in blindness and low vision by 2020 [14]. Vision loss contributes significantly to falls, fractures and restrictions in mobility [15], and to increased hospital length of stay and post-discharge requirements for rehabilitative care [16]. Severe bilateral visual impairments are associated with an increased risk of all-cause mortality and cardiovascular disease-related mortality [17], and are regarded as an independent predictor of mortality [17,18].

Preventive health examinations are an important health promotion strategy [19-22]. They can help to identify diseases at an early stage, postpone the development of subsequent adverse outcomes, and significantly save healthcare resources and lives [19]. Recent research from the United States suggests that greater use of clinical preventive services can save more than two million life-years annually [20]. In Japan, Hozawa et al. reported that mortality rates are at least 26% lower among those undergoing health check-ups than those who do not [21]. In Taiwan, Deng et al. reported that for hypertension patients who attended a health examination program, over NT$34,570 in healthcare costs were saved, and life-spans were increased by 128 days [22].

Equity of access to health care is an important factor in priority setting of a health care system [23,24]. Inequity in access to preventive health services has been shown to be closely related to differences in age, family income, gender, race/ethnicity, urban/rural residence, severity of disability, and education level [25-29].

In Taiwan, the government inaugurated the National Health Insurance (NHI) program in March 1995 to provide compulsory universal health care coverage including medical care services and preventive health services. To date, the NHI enrolls over 99.9% of the Taiwanese population [30] and has contracts with over 92% of all medical providers [31]. Since the launch of the NHI, investigations have reported there to be significant improvements in terms of equity of access to health care, greater financial risk protection, and the geographical distributions of physicians [32-35].

The overall utilization rate of adult health examinations in Taiwan has been reported to be 33.3% to 40.72% [36,37], and 46.8% in the elderly [19]. For the whole disabled population, the utilization rate has been reported to be 15.8% [13]. Although the utilization rate in the disabled population is much lower, disabilities are usually not factored into most studies on equity. To reduce the barriers to preventive health services and encourage health checks for the visually disabled population, it is necessary to obtain evidence from large-scale investigations regarding the associated factors of utilization of the preventive health services. Accordingly, the aim of this study was to examine the factors related to the utilization of health examinations by the visually disabled population. Identifying the barriers that prevent visually disabled people from participating in health examinations may help the authorities to conceive feasible strategies for this marginalized population.

## Methods

### Preventive health services in Taiwan

To promote the health of all people in Taiwan, the government has provided free preventive health services since 1995. These services are provided free (only a registration fee is required) to: (1) those aged 40–64 years once every three years; (2) those aged 65 years and over once a year; and (3) those with poliomyelitis aged 35 and over once a year. A registration fee of up to US$3 may be required for people with no disabilities, although this is waived for people with disabilities. The health examination includes a physical examination, health education guidance, blood tests, and urinalysis.

### Study population

This study focused on adults aged over 40 years with visual disabilities. Visual disability, like all other formally issued disabilities in Taiwan, was authenticated via a strict administrative process. Patients with poor vision were examined and certified at ophthalmology clinics, and then approved by the Ministry of the Interior (MOI), Taiwan.

### Data sources

Three different nationwide datasets were used including the Disability Registration from the MOI 2008 (with access to demographic characteristics and severity of disabilities), the Health Insurance Medical Claims from the National Health Research Institutes 2008 (with access to income status and health status), and the Health Prevention Services File from the Bureau of Health Promotion from 2006 to 2008 (with access to health examination utilization status). This study has been approved by the research ethics committee in China Medical University and Hospital (IRB No. CMU-REC-101-012).

### Relevant variables

The demographic characteristics including gender, age, education level, marital status, aboriginal status (yes vs. no), and level of urbanization of residential area (district or township) were recorded. The definition of level of urbanization was designed by Liu et al. [38] and has been broadly utilized in relevant research. Urbanization was classified into 8 levels for all residential townships in Taiwan, with level 1 being the most urbanized areas and level 8 being the least urbanized areas. The severity of disability was classified as mild, moderate, severe and very severe.

Income status included a low-income household status (yes vs. no) and levels of premium-based monthly salary (PBMS). The low-income household status was defined as a household per capita income of below the minimum cost of living for that residential area. The levels of PBMS were the monthly income levels reported to the Bureau of National Health Insurance as the basis for insurance premium collection and are often used as the index for personal income. Those who are members of a family but without employment are enrolled as insured dependents in the National Health Insurance program.

Health status included catastrophic illness/injury (yes vs. no) and the presence of relevant chronic illnesses (including cancer, endocrine and metabolic diseases, mental disorders, diseases of the nervous system, diseases of the circulatory system, diseases of the respiratory system, diseases of the digestive system, diseases of the genitourinary system, diseases of the musculoskeletal system and connective tissue, disorders of the eye and adnexa, infectious diseases, congenital anomalies, diseases of the skin and subcutaneous tissue, diseases of the blood and blood-forming organs, and diseases of the ear and mastoid process). Whether or not each subject had utilized a health examination was also recorded (yes vs. no).

### Statistical analysis

The chi-square test was used for descriptive analysis of the variables, with a *p* value of less than 0.05 being considered statistically significant. Multivariate logistic regression analysis was subsequently used to examine the influencing factors on the utilization of health examinations. The independent variables included demographic characteristics, income status, health status, and severity of disability, and the use of health examinations (yes vs. no) was the dependent variable.

All analyses were performed using SAS statistical software (version 9.1 for Windows; SAS Institute, Inc., Cary, NC, USA).

## Results

In total, 47,812 (23,450 female, 49.05%; 24,362 male, 50.95%) visually disabled people were enrolled. The overall health examination utilization rate was 16.16% (females 17.12% vs. males 15.25%, *p* < 0.001), while the benchmark data for the general population during the study period was 33.3% to 40.72% [36,37]. In terms of age, over 70% of the cohort was over 60 years of age (Table 1). With regards to urbanization level, less visually disabled people lived in Level 4 and Level 8 areas (< 10%). Those who lived in Level 1 areas had a relatively lower utilization rate. In terms of PBMS, most subjects were in the insured dependent group (38.68%), followed by those with a PBMS of 16500–22800 (29.96%) and < 15840 (20.26%) New Taiwan dollars (NTD), respectively. These three subgroups constituted 88.9% of the whole population, showing that the majority of the cohort either had limited income or were unemployed. The insured dependent subgroup and the subgroup with the highest PBMS (PBMS NTD 48200–57800) reported lower utilization rates (around 13%) than the overall utilization rate (Table 1).

**Table 1:** Characteristics and Chi-square analysis of the health examination utilization in the visually disabled population

					**Used**		**Did not use**		**χ2**
**Variables**			**N = 47812**	**%**	**n**_**1**_ **= 7728**	**%**	**n**_**2**_ **= 40084**	**%**	**p-value**
Overall utilization rate						16.16			
Gender									<.001*
	Female		23450	49.05	4014	17.12	19436	82.88	
	Male		24362	50.95	3714	15.25	20648	84.75	
Age									<.001*
	40-44		1555	3.25	264	16.98	1291	83.02	
	45-49		3264	6.83	650	19.91	2614	80.09	
	50-54		4092	8.56	908	22.19	3184	77.81	
	55-59		4935	10.32	1184	23.99	3751	76.01	
	60-64		4790	10.02	1225	25.57	3565	74.43	
	65-69		6020	12.59	843	14	5177	86	
	≧70		23156	48.43	2654	11.46	20502	88.54	
Education									<.001*
	Primary school and below		29471	61.64	4518	15.33	24953	84.67	
	Middle school		4534	9.48	847	18.68	3687	81.32	
	High school		4729	9.89	855	18.08	3874	81.92	
	Post-secondary education		2649	5.54	438	16.53	2211	83.47	
	Unknown		6429	13.45	1070	16.64	5359	83.36	
Marital status									<.001*
	Married		28765	60.16	4890	17	23875	83	
	Single		3486	7.29	560	16.06	2926	83.94	
	Divorced or widowed		2265	4.74	369	16.29	1896	83.71	
	Unknown		13296	27.81	1909	14.36	11387	85.64	
Aboriginal status									0.000*
	Yes		651	1.36	138	21.2	513	78.8	
	No		47161	98.64	7590	16.09	39571	83.91	
Urbanization level									<.001*
	Level 1		5123	10.71	685	13.37	4438	86.63	
	Level 2		9005	18.83	1509	16.76	7496	83.24	
	Level 3		6367	13.32	1014	15.93	5353	84.07	
	Level 4		3740	7.82	653	17.46	3087	82.54	
	Level 5		6997	14.63	1179	16.85	5818	83.15	
	Level 6		5340	11.17	896	16.78	4444	83.22	
	Level 7		6527	13.65	1070	16.39	5457	83.61	
	Level 8		4713	9.86	722	15.32	3991	84.68	
Premium-based monthly salary (NT$)									<.001*
	Dependent		18495	38.68	2386	12.9	16109	87.1	
	<15,840		9688	20.26	1494	15.42	8194	84.58	
	16,500-22,800	14324	29.96	2675	18.67	11649	81.33		
	24,000-28,800	1499	3.14	351	23.42	1148	76.58		
	30,300-36,300	1299	2.72	328	25.25	971	74.75		
	38,200-45,800	1954	4.09	422	21.6	1532	78.4		
	48,200-57,800	553	1.16	72	13.02	481	86.98		
Low-income household									0.185
	Yes		1470	3.07	256	17.41	1214	82.59	
	No		46342	96.93	7472	16.12	38870	83.88	
Catastrophic illness/injury									<.001*
	Yes		4240	8.87	539	12.71	3701	87.29	
	No		43572	91.13	7189	16.5	36383	83.5	
Relevant chronic disease									
	Cancer								<.001*
		Yes	2278	4.76	288	12.64	1990	87.36	
		No	45534	95.24	7440	16.34	38094	83.66	
	Endocrine and metabolic disorder								<.001*
		Yes	21852	45.7	4326	19.8	17526	80.2	
		No	25960	54.3	3402	13.1	22558	86.9	
	Mental disorder								<.001*
		Yes	11338	23.71	2287	20.17	9051	79.83	
		No	36474	76.29	5441	14.92	31033	85.08	
	Diseases of the nervous system								<.001*
		Yes	6991	14.62	1360	19.45	5631	80.55	
		No	40821	85.38	6368	15.6	34453	84.4	
	Diseases of the circulatory system								<.001*
		Yes	26891	56.24	4892	18.19	21999	81.81	
		No	20921	43.76	2836	13.56	18085	86.44	
	Diseases of the respiratory system								<.001*
		Yes	12016	25.13	2385	19.85	9631	80.15	
		No	35796	74.87	5343	14.93	30453	85.07	
	Diseases of the digestive system								<.001*
		Yes	18885	39.5	3774	19.98	15111	80.02	
		No	28927	60.5	3954	13.67	24973	86.33	
	Diseases of the genitourinary system								0.272
		Yes	3465	7.25	583	16.83	2882	83.17	
		No	44347	92.75	7145	16.11	37202	83.89	
	Diseases of the musculoskeletal system and connective tissue								<.001*
		Yes	19214	40.19	3895	20.27	15319	79.73	
		No	28598	59.81	3833	13.4	24765	86.6	
	Diseases of the eyes and adnexa								<.001*
		Yes	21623	45.23	4075	18.85	17548	81.15	
		No	26189	54.77	3653	13.95	22536	86.05	
	Infectious disease								<.001*
		Yes	2599	5.44	507	19.51	2092	80.49	
		No	45213	94.56	7221	15.97	37992	84.03	
	Congenital anomalies								<.001*
		Yes	986	2.06	208	21.1	778	78.9	
		No	46826	97.94	7520	16.06	39306	83.94	
	Diseases of skin and subcutaneous tissue								<.001*
		Yes	5602	11.72	1090	19.46	4512	80.54	
		No	42210	88.28	6638	15.73	35572	84.27	
	Diseases of blood and blood-forming organs								<.001*
		Yes	2632	5.5	511	19.41	2121	80.59	
		No	45180	94.5	7217	15.97	37963	84.03	
	Diseases of the ear and mastoid process								<.001*
		Yes	5135	10.74	1060	20.64	4075	79.36	
		No	42677	89.26	6668	15.62	36009	84.38	
Severity of disability									<.001*
	Mild		15622	32.67	2993	19.16	12629	80.84	
	Moderate		14050	29.39	2421	17.23	11629	82.77	
	Severe		18138	37.94	2314	12.76	15824	87.24	
	Very severe	2	0	0	0	2	100		

*p < 0.05.

Around three percent (3.07%) of the population were classified as belonging to low-income households, however the health examination utilization rate in this subgroup was higher than for those who were not classified as being in low income households (17.41% versus 16.12%). With regards to aboriginal status, 1.36% of the population was classified as being aborigines, and this group had a higher utilization rate than non-aboriginal people. In terms of education level, 61.64% of the population had a level of primary school or below and they had a significantly lower utilization rate. In terms of marital status, most of the population was married (60.16%), and this subgroup had a higher utilization rate than the other subgroups. Those who had any catastrophic illness/injury (8.87%) had a significantly lower utilization rate (12.71%). Similarly, those who suffered from cancer also had a significantly lower utilization rate (12.64%). Those with chronic diseases had a higher utilization rate than those without chronic diseases (Table 1). In terms of disability severity, those with severe and very severe disabilities had significantly lower utilization rates.

### Factors associated with the utilization of preventive health services

Multivariate logistic regression analysis revealed the likelihood of utilization to be significantly lower in males compared to females after controlling for other variables (Table 2). Compared with the 40–44 years subgroup, the utilization probability in the 65–69 and ≥ 70 years subgroups were 39% and 52% lower, respectively (OR = 0.61 and 0.48, both *p* < 0.001). In comparison to Level 1 urbanization areas, the probabilities of utilization by residents in all other levels were significantly higher. In comparison to the PBMS NTD < 15840 subgroup, the probability of utilization in the insured dependent subgroup was significantly lower (OR = 0.92, 95% CI: 0.85-0.99, *p* = 0.035), and that of the top level subgroup (NTD 48200–57800) was even lower (OR = 0.63, 95% CI: 0.48-0.81, *p* = 0.001). Those with catastrophic illness/injury had a much lower utilization probability (OR = 0.64, 95% CI: 0.56-0.74, *p* < 0.001). In terms of chronic diseases, after controlling for other variables, only the subgroup with diseases of the genitourinary system had a significantly lower probability of utilization (OR = 0.83, 95% CI: 0.75-0.92, *p* < 0.001), whereas those with most other chronic diseases had either comparable or higher probabilities of utilization. In comparison to those with mild disabilities, the probability of utilization in those with moderate disabilities was 8% lower, and 21% lower in those with severe and very severe disabilities (OR = 0.79, 95% CI: 0.74-0.84, *p* < 0.001).

**Table 2:** Logistic regression analysis of the health examination utilization probability in the visually disabled population

		**Unadjusted Model**				**Adjusted Model**			
**Variable**		**OR**	**95% CI**		**p-value**	**OR**	**95% CI**		**p-value**
Gender									
	Female	-	-	-	-	-	-	-	-
	Male	0.87	0.83	0.91	<.001*	0.88	0.84	0.93	<.001*
Age									
	40-44	-	-	-	-	-	-	-	-
	45-49	1.22	1.04	1.42	0.015*	1.14	0.97	1.34	0.117
	50-54	1.40	1.20	1.62	<.001*	1.21	1.04	1.42	0.016*
	55-59	1.54	1.33	1.79	<.001*	1.27	1.09	1.49	0.002*
	60-64	1.68	1.45	1.95	<.001*	1.34	1.14	1.56	<.001*
	65-69	0.80	0.69	0.93	0.003*	0.61	0.51	0.71	<.001*
	≧70	0.63	0.55	0.73	<.001*	0.48	0.42	0.56	<.001*
Education									
	Primary school and below	-	-	-	-	-	-	-	-
	Middle school	1.27	1.17	1.38	<.001*	1.04	0.95	1.13	0.447
	High school	1.22	1.13	1.32	<.001*	1.02	0.93	1.11	0.713
	Post-secondary education	1.09	0.98	1.22	0.1	1.06	0.94	1.19	0.372
	Unknown	1.10	1.03	1.19	0.009*	1.07	0.99	1.16	0.074
Marital status									
	Married	-	-	-	-	-	-	-	-
	Single	1.07	0.97	1.18	0.164	1.00	0.91	1.11	0.958
	Divorced or widowed	1.02	0.88	1.17	0.819	0.98	0.84	1.14	0.767
	Unknown	0.88	0.79	0.97	0.011*	0.85	0.76	0.94	0.003*
Aboriginal status									
	No	-	-	-	-	-	-	-	-
	Yes	1.40	1.16	1.70	0.001*	1.18	0.97	1.44	0.107
Urbanization level									
	Level 1	-	-	-	-	-	-	-	-
	Level 2	1.30	1.18	1.44	<.001*	1.30	1.17	1.44	<.001*
	Level 3	1.23	1.11	1.36	<.001*	1.27	1.14	1.42	<.001*
	Level 4	1.37	1.22	1.54	<.001*	1.38	1.22	1.56	<.001*
	Level 5	1.31	1.19	1.45	<.001*	1.41	1.26	1.57	<.001*
	Level 6	1.31	1.17	1.46	<.001*	1.40	1.25	1.58	<.001*
	Level 7	1.27	1.15	1.41	<.001*	1.46	1.30	1.63	<.001*
	Level 8	1.17	1.05	1.31	0.006*	1.27	1.12	1.44	<.001*
Premium based monthly salary (NT$)									
	<15,840	-	-	-	-	-	-	-	-
	Dependent	0.81	0.76	0.87	<.001*	0.92	0.85	0.99	0.035*
	16,500-22,800	1.26	1.18	1.35	<.001*	1.16	1.07	1.26	<.001*
	24,000-28,800	1.68	1.47	1.91	<.001*	1.17	1.01	1.34	0.034*
	30,300-36,300	1.85	1.62	2.12	<.001*	1.21	1.04	1.39	0.013*
	38,200-45,800	1.51	1.34	1.71	<.001*	1.11	0.98	1.27	0.11
	48,200-57,800	0.82	0.64	1.06	0.128	0.63	0.48	0.81	0.001*
Low-income household									
	No	-	-	-	-	-	-	-	-
	Yes	1.10	0.96	1.26	0.186	1.03	0.89	1.21	0.677
Catastrophic illness/injury									
	No	-	-	-	-	-	-	-	-
	Yes	0.74	0.67	0.81	<.001*	0.64	0.56	0.74	<.001*
Relevant chronic disease									
	Cancer	0.74	0.65	0.84	<.001*	1.14	0.95	1.37	0.172
	Endocrine and metabolic disorder	1.64	1.56	1.72	<.001*	1.22	1.15	1.30	<.001*
	Mental disorder	1.44	1.37	1.52	<.001*	1.16	1.09	1.24	<.001*
	Diseases of the nervous system	1.31	1.23	1.40	<.001*	0.99	0.92	1.06	0.697
	Diseases of the circulatory system	1.42	1.35	1.49	<.001*	1.17	1.10	1.24	<.001*
	Diseases of the respiratory system	1.41	1.34	1.49	<.001*	1.21	1.14	1.29	<.001*
	Diseases of the digestive system	1.58	1.50	1.66	<.001*	1.24	1.17	1.31	<.001*
	Diseases of the genitourinary system	1.05	0.96	1.16	0.272	0.83	0.75	0.92	<.001*
	Diseases of the musculoskeletal system and connective tissue	1.64	1.56	1.73	<.001*	1.38	1.31	1.46	<.001*
	Diseases of the eyes and adnexa	1.43	1.36	1.50	<.001*	1.13	1.07	1.19	<.001*
	Infectious disease	1.28	1.15	1.41	<.001*	1.07	0.96	1.20	0.194
	Congenital anomalies	1.40	1.20	1.63	<.001*	1.10	0.94	1.29	0.255
	Diseases of skin and subcutaneous tissue	1.30	1.21	1.39	<.001*	1.08	1.00	1.17	0.053
	Diseases of blood and blood-forming organs	1.27	1.15	1.40	<.001*	1.05	0.95	1.17	0.335
	Diseases of the ear and mastoid process	1.41	1.31	1.51	<.001*	1.10	1.02	1.19	0.015*
Severity of disability									
	Mild	-	-	-	-	-	-	-	-
	Moderate	0.88	0.83	0.93	<.001*	0.92	0.87	0.98	0.011*
	Severe + Very severe	0.62	0.58	0.66	<.001*	0.79	0.74	0.84	<.001*

*p < 0.05.

Further, those with a low-income household status, aboriginal status, and lower education level, which are traditionally regarded as being disadvantaged subgroups, were found to have no significant differences in the probabilities of utilizing health examinations.

## Discussion

This is the first comprehensive nationwide study to report the preventive health examination usage status in the visually disabled population in Taiwan. The findings show that the rate of using preventive health examinations in this cohort is extremely low (16.16%) compared to the general population who were not visually impaired (33.3% to 40.72%) [36,37]. In terms of age, over 60% of the cohort were aged 65 years or over. Chang et al. reported that the preventive health service utilization rate in the elderly in Taiwan is 46.8% [19], and another study reported that in aging Chinese Canadians, the rate is 76% [39]. These data suggest that most visually disabled people are elderly, and that the rate of using preventive health examinations in this population is very low. As indicated previously by evidence from different countries, an increased usage of preventive health examinations may improve health, reduce mortality and lower health care costs [20-22]. Therefore, it is imperative to enhance the utilization of preventive health examinations and improve the health status of this population.

Male gender, regardless of age, was significantly associated with a lower health examination usage, which is similar to previous reports [13,21,26]. In Taiwan, men still play the traditional role of familial financial support even if they have visual disabilities [40]. This could be ameliorated by advocating on-site health checks in companies through proper planning. In addition, men tend to pay less attention to their own healthcare in Taiwan [41]. Further health education and encouraging couples to attend examinations may be helpful in this regard.

Of those who were found to have lower preventive health examination utilization, certain subgroups could be considered to be disadvantaged with regards to healthcare resources, possibly due to lower access. They included the insured dependent subgroup, the elderly population, and those with a moderate or worse severity of disability. These subgroups share certain common characteristics. First, they are typical disadvantaged groups who are unemployed, with illnesses or senility, and need financial or transportation assistance. Second, they lack the personal ability to seek health services. Third, they may be reluctant to become a burden on their family. Several recommendations to enhance health examination utilization in these marginalized subgroups have been reported. For the disabled, transportation is an important barrier to access to health services in addition to financial constraints and communication difficulties [42,43]. Free transportation is widely available in Taiwan [44], however it is used less frequently by people with disabilities. In addition, patient-family support groups have been developed for patients with cancer or disabilities, and have been shown to be helpful in improving adjustment and self-reliance [45,46]. To boost the utilization rate in this subgroup, healthcare authorities may need to address these points by providing more resources and initiative services.

People dwelling in the least urbanized regions such as the offshore islands and remote areas, aborigines, those with a low income, and those with lower education levels are traditionally considered to be disadvantaged groups and are expected to experience worse healthcare equity. However, the utilization rates in these groups were not lower in this study. Mobile health services and special programs initiated by the government to provide healthcare services to the remote and mountainous areas provide good healthcare access and may be the reason for the comparable utilization rates. In addition, the costs for the low-income households are covered by the Taiwan welfare system for co-payments per visit and National Health Insurance monthly premiums, and this may have played a role in enhancing preventive health service utilization in this disadvantaged group. Finally, those with a lower education level unexpectedly had a comparable utilization, which implies that, in Taiwan, other demographic factors such as age, income, or health status may be more closely associated with inequity.

Other subgroups that were found to have lower utilization rates were not considered to be disadvantaged groups, and may have had more health service alternatives. This includes the subgroup dwelling in the most urbanized region (Level 1), and the subgroup reporting the highest income (the subgroup with the top-ranked PBMS). These results seem to be in contrast to other published reports; however they represent the subjects with a higher socio-economic status who may have more options for better self-paid preventive health services. In addition, these subjects might belong to health clubs which provide top-level health check programs, and therefore forego the free lower level preventive health checks provided by the National Health Insurance program.

Those with catastrophic illnesses and those with chronic systemic diseases of the genitourinary system were the two subgroups that had the lowest usage. However, these subjects would already have their preventive health service needs satisfied by scheduled regular check-ups at specialist clinics due to the underlying illness. All co-payments for such health services are exempt due to the status of having a catastrophic illness, and thus these patients would most likely not require the free standard preventive health checks provided by the National Health Insurance program.

There are some limitations to this study. First, utilization of healthcare services is closely related to understanding the health service and social welfare systems, and this can be challenging for those who are unfamiliar with these systems. Second, this is a secondary dataset research based on three different data files. Factors such as health beliefs and family history of illnesses may influence the utilization of health examinations, however these factors were not included in the datasets. In addition, PBMS but not true income data was used for analysis, which may not represent the true income levels. Third, only those aged 40 or above were included in this study and extrapolation of the results to younger age groups would be inappropriate.

## Conclusion

The overall preventive health examination utilization rate in the visually disabled population is very low in Taiwan. The subgroups with lower utilization included male gender, elderly subjects aged 65 years and above, subjects living in the most urbanized regions, dependent subjects, subjects with a higher income level, subjects with catastrophic illnesses and genitourinary system diseases, and subjects with moderate or more severe disabilities. These findings have important implications for the healthcare policy makers who seek to reduce health disparity and enhance equity of healthcare for the visually disabled population. More resources should be allocated to address the issue of inequity in accessing healthcare in Taiwan.

## Abbreviations

MOI: The Ministry of the Interior; PBMS: Premium-based monthly salary.

## Competing interests

The authors declare that they have no competing interests.

## Authors' contributions

WCT and PTK contributed to the conception and design of the study and to statistical analysis. YHH and WCT wrote the first draft of the manuscript. All authors participated in the interpretation of data for important intellectual content, and revised and approved the final version of the manuscript.

## Pre-publication history

The pre-publication history for this paper can be accessed here:

http://www.biomedcentral.com/1472-6963/13/509/prepub
